# Low friction layer in sanitary products for reduced skin irritation

**DOI:** 10.1038/s41598-025-17226-4

**Published:** 2025-09-29

**Authors:** Toshiaki Nishi, Kenta Matsui, Rina Ito, Mihoko Kuramochi, Masaya Fujita, Takeshi Yamaguchi

**Affiliations:** 1 Graduate School of Engineering, Tohoku University, 6-6-01, Aoba, Aramaki-Aza, Aoba-Ku, Senda, Miyagi 980-8579 Japan; 2 Daio Paper Corporation, 2-10-2 Fujimi, Chiyoda-ku, Tokyo, 102-0071 Japan; 3 Graduate School of Biomedical Engineering, Tohoku University, 6-6-01, Aoba, Aramaki-Aza, Aoba-ku, Sendai, Miyagi 980-8579 Japan

**Keywords:** Sanitary napkin, Artificial skin, Friction, Strain, Digital image correlation method, Skin models, Biomedical engineering, Quality of life, Design, synthesis and processing

## Abstract

Sanitary napkins are essential during menstruation, but they can sometimes cause skin irritation due to friction. In this study, we propose a novel design: introducing a low-friction layer within the bulk of a sanitary napkin. Friction tests were conducted between an artificial skin block and sanitary napkin specimens with and without a low-friction layer. The strain distribution on the lateral side of the artificial skin block was experimentally measured. The friction coefficient and strain decreased when the low-friction layer was applied under loads greater than 1.96 N. The strain increased with normal load. Because lower strain on the skin correlates with reduced skin irritation, the use of a low-friction layer in sanitary napkins is expected to reduce skin irritation, particularly under high contact pressure.

## Introduction

Menstruation is a biological process that begins around the ages of 12–13 and continues throughout a woman’s reproductive years, typically lasting until the ages of 45–50^1^. During menstruation, menstrual blood is discharged involuntarily^2^. On average, women experience menstruation approximately 1,368 to 2,192 times in their lifetime, and each menstrual cycle lasts between 3 and 7 days^3^. Menstruation significantly influences women’s lives, making comfort during this period an important factor for improving their quality of life^4^. Sanitary items, such as sanitary napkins and tampons, are indispensable for menstrual life. Sanitary napkins are disposable hygiene items categorized as technical textiles because they contain functional textile materials^5^. The market for sanitary napkins is large^6^ and is expected to remain large until new technologies that offer ease of use, accessibility, hygiene, and comfort^5^.

Sanitary napkins are manufactured as a layered structure to satisfy different properties such as absorbency, leak resistance, and comfort^5^. The layers include woven and film materials. The top layer that contacts the body is the top sheet. The top sheet can be a polyethylene film or nonwoven polypropylene^5,7^. The absorbent layer is usually made of wood pulp and superabsorbent polymers. It is manufactured using airlaid technology and is available in various shapes. The bottom layer or back sheet is usually an impermeable film^6–13^. The absorbent layer is made of nonwoven fabrics, which are simple and fast to manufacture, absorb large amounts of liquid, and provide user comfort^5,7,10–13^.

Given the thin and sensitive nature of the skin in the private areas^14^, minimizing irritation is an important factor in sanitary napkin design, in addition to the absorbent, sealing, and breathability performances^15^. Many women feel “rubbing” as an unpleasant symptom^5^ when wearing sanitary napkins. Therefore, “low skin irritation” is a key consideration when purchasing sanitary napkins. However, there are few studies on the cause of “rubbing.” Reducing the strain on the skin caused by the stress acting on it is necessary to reduce skin irritation caused by friction between sanitary napkins and the skin while walking. Sugiyama et al. experimentally estimated, using a human lower limb model, that the sliding distance and maximum contact pressure in one stride while walking were 0.176 mm and 2.6 kPa, respectively^16^. Takimoto reported that the maximum contact pressure on the hip while sitting was 13.3 kPa^17^. Prolonged pressure on the skin, which is not limited to the private parts, can lead to skin damage, such as ulcers and bedsores^18,19^. Studies have shown that high-pressure and shear forces increase the risk of skin injuries^20^. Therefore, reducing the pressure and shear on the skin is a logical approach to preventing damage, including irritation to the delicate skin in private parts, when using sanitary napkins.

Generally, the layers of the sanitary napkin are bonded together. The shear and friction forces are distributed via these bonded interfaces. However, from an engineering standpoint, what if the interfaces inside sanitary napkins are not bonded? The irritation to the skin could be reduced by minimizing the transmission of shear and friction forces via the interfaces in the sanitary napkin. The interfaces should remain unchanged because they do not prevent the flow of menstrual blood from the top sheet to the absorbent layer. Thus, to achieve low irritation to the skin, a nonbonded interface should be inserted between the absorbent layer and the bottom sheet inside the sanitary napkin.

This study investigates the effect of such a low-friction layer inside a sanitary napkin on friction-induced strain in an artificial skin block, which is considered a mechanical factor potentially contributing to skin irritation. The friction test between the artificial skin block and the sanitary napkin was conducted in accordance with ethical principles. The skin irritation was quantified based on the maximum strain in the artificial skin block. The effect of the normal load between the skin and napkin on the maximum strain was also investigated.

## Results and discussion

### Friction behavior

**Fig. 1:**
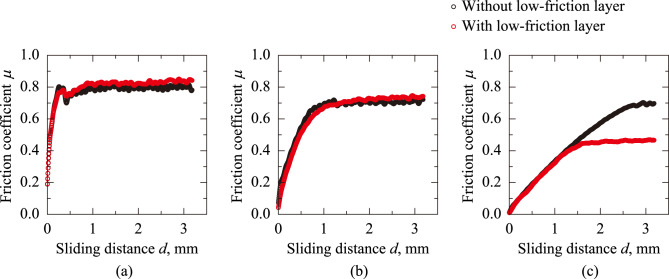
Friction coefficient *μ* plotted against sliding distance *d* for normal loads *W* = 0.490, 1.96, and 7.84 N for sanitary napkin specimens with and without a low-friction layer.

Fig. 1 shows the friction coefficient *μ* plotted against the sliding distance *d* for normal loads *W* = 0.490, 1.96, and 7.84 N, comparing the sanitary napkin specimen with and without the low-friction layer. Regardless of *W* or the low-friction layer, *μ* increased and reached a constant value. The smaller the *W*, the faster *μ* reached its saturation point. While the saturated value of *μ* was nearly the same at *W* = 0.490 and 1.96 N for both specimens, the value was lower at *W* = 7.84 N for the sanitary napkin specimen with the low-friction layer. The mean value of *μ* at *d* = 2.65–3.18 mm was defined as the dynamic friction coefficient *μ*_d_.

**Fig. 2.**
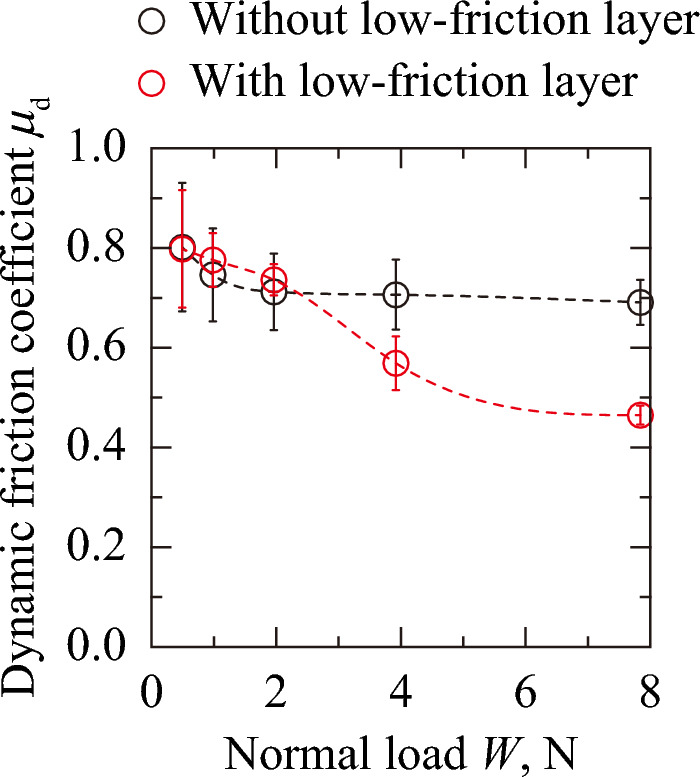
Relationship between the dynamic friction coefficient and normal load for sanitary napkin specimens with and without a low-friction layer.

Fig. 2 shows the relationship between *μ*_d_ and *W* for the sanitary napkin specimens with and without the low-friction layer. The error bars represent the standard deviation of each condition. For the sanitary napkin specimen with the low-friction layer, *μ*_d_ decreased with *W*. For the sanitary napkin specimen without the low-friction layer *μ*_d_ also decreased with *W* at *W* = 0.490–1.96 N but remained constant at *W* = 1.96–7.84 N. This result indicates that the low-friction layer reduced friction at higher normal loads (*W* = 1.96–7.84 N). Assuming Hertzian contact between the artificial skin block and top sheet with a fixed Poisson’s ratio of 0.5, the contact area is proportional to *W* to the power of 1/3 for point contact and 1/2 for line contact. Accordingly, the adhesion term is proportional to *W* to the power of –2/3 and –1/2, respectively. Therefore, it is reasonable that *μ*_d_ tends to decrease with increasing *W*. However, the *W* dependence of *μ*_d_ is considered to depend on the relative contributions of the adhesion and hysteresis terms, as well as on possible changes in Poisson’s ratio during compression. It is considered here that the *W* dependence of *μ*_d_ differs between the interfaces of the artificial skin block/top sheet and the absorbent layer/bottom sheet. Here, the range of contact pressure in practical use is from 2.6 kPa^16^ to 13.3 kPa^17^, which approximately corresponds to *W* = 0.98–24.4 N in this experimental system. Therefore, applying the low-friction layer is expected to reduce skin irritation within the practical pressure range. Furthermore, no signs of excessive slippage or instability were observed during the experiments, indicating that the reduction in friction was moderate and did not impair functional performance.

### Strain distribution during friction

**Fig. 3:**
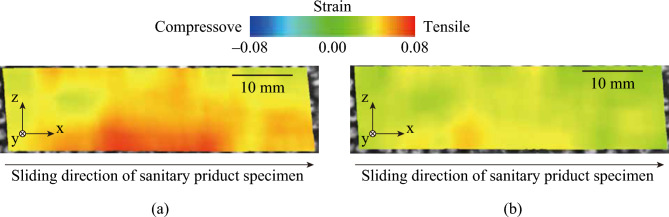
Distribution of the major principal strain *ε*_1_ on the lateral side of the artificial skin specimen with and without the low-friction layer at a sliding distance *d* = 3.18 mm and normal load *W* = 7.84 N

**Fig. 4:**
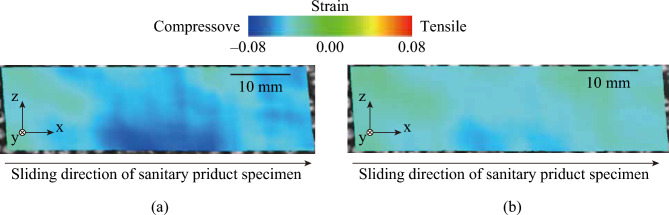
Distribution of the minor principal strain *ε*_2_ on the lateral side of the artificial skin specimen with and without a low-friction layer at the sliding distance *d* = 3.18 mm and normal load *W* = 7.84 N

Fig. 3 shows the distribution of the major principal strain *ε*_1_ on the lateral side of the artificial skin block for the sanitary napkin specimen with and without the low-friction layer at a sliding distance *d* = 3.18 mm and a normal load *W* = 7.84 N. The green area represents the region where *ε*_1_ = 0.00, and the blue and red areas represent compressive and tensile stresses, respectively. Regardless of the sanitary napkin specimens, tensile stress (*ε*_1_ > 0) was observed in the entire specimen, and *ε*_1_ was maximized at the center of the lower half in each specimen. Note that *ε*_1_ was maximized at the bulk material interface. This aligns with Hertz contact theory^21^, which states that strain is maximized in the bulk of a material facing another object, not at the interface^22^, making this result reasonable. The minor principal strain *ε*_2_ under the same conditions (Fig. 3) is shown in Fig. 4. Regardless of the sanitary napkin specimens, compression strain was observed in the entire specimen and *ε*_2_ was smallest at the center of the lower half in each specimen. For both *ε*_1_ and *ε*_2_, the range of strain was smaller in the sanitary specimen with the low-friction layer than in the specimen without it.

**Fig. 5:**
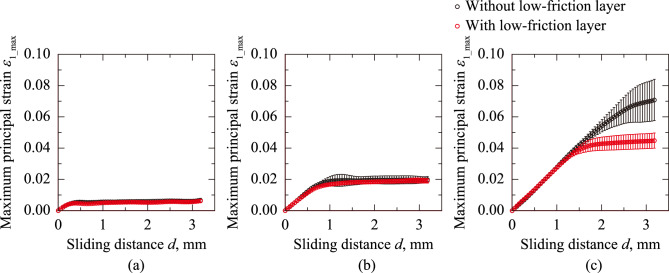
Maximum principal strain *ε*_1_max_ plotted against sliding distance *d* at normal load *W* = 0.490, 1.96, and 7.84 N for sanitary napkin specimens with and without a low-friction layer

**Fig. 6:**
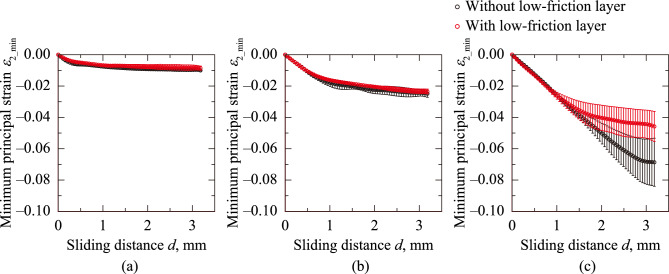
Minimum principal strain *ε*_2_min_ plotted against sliding distance *d* at normal load *W* = 0.490, 1.96, and 7.84 N for sanitary napkin specimens with and without a low-friction layer

Studies have shown that the tactile sense is sensitive not only to friction^23,24^ but also to the strain in the skin^25–27^. Thus, quantifying skin irritation based on the maximum strain is crucial. To determine the maximum stain in tensile and compression, the maximum major principal strain *ε*_1_max_ and the minimum minor principal strain *ε*_2_min_ are defined as the maximum and minimum values of *ε*_1_ and *ε*_2_ at each phase, respectively. Fig. 5 plots *ε*_1_max_ against the sliding distance *d* at *W* = 0.490, 1.96, and 7.84 N for the sanitary napkin specimen with and without the low-friction layer. Regardless of the sanitary napkin specimens and *W* condition, *ε*_1_max_ increased and reached constant values. The larger the *W*, the wider the range of *d*, where *ε*_1_max_ indicated an increasing trend. The relationship between *ε*_2_min_ and *d* at *W* = 0.490, 1.96, and 7.84 N for the sanitary napkin specimen with and without the low-friction layer is shown in Fig. 6. For all conditions, *ε*_2_min_ decreased with *d*. The decreasing rate of *ε*_2_min_ was high, particularly after the sliding began. Similar to the *ε*_1_max_–*d* curves (Fig. 5), as *W* increased, the range of *d* expanded, where the decreasing rate remained high. These results indicate that the strain accumulated during the initial sliding phase reaches a steady state as the energy is dissipated through friction. The constant values of *ε*_1_max_ and *ε*_2_min_ at *W* = 7.84 N were changed by applying the low-friction layer to the sanitary napkin specimen (Figs. 5(c) and 6(c)). This indicates that the low-friction layer enhanced the energy dissipation during friction. However, this effect can be limited to the sliding distance *d* corresponding to the allowable movement of the low-friction layer.

**Fig. 7:**
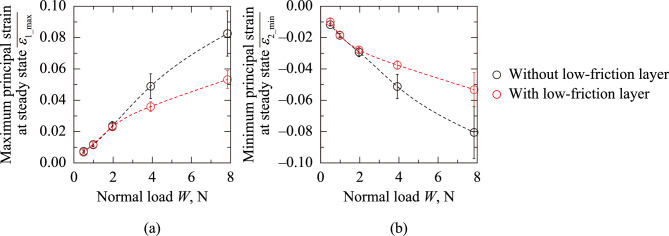
Influence of normal load *W* on maximum $\overline{{\varepsilon_{{{\text{1\_max}}}} }}$ and minimum principal strains at steady state $\overline{{\varepsilon_{{{\text{2\_min}}}} }}$ in sanitary napkin specimens with and without a low-friction layer

To quantify skin irritation under steady state conditions, the maximum and minimum principal strains at steady state $\overline{{\varepsilon_{{{\text{2\_min}}}} }}$ were defined as the mean values of *ε*_1__$\overline{{\varepsilon_{{{\text{1\_max}}}} }}$_max_ and *ε*_2_min_ at *d* = 2.65–3.18 mm, respectively. Fig. 7 shows the influence of *W* on $\overline{{\varepsilon_{{{\text{1\_max}}}} }}$ and $\overline{{\varepsilon_{{{\text{2\_min}}}} }}$ for sanitary napkin specimens with and without a low-friction layer. The absolute value of $\overline{{\varepsilon_{{{\text{1\_max}}}} }}$ and $\overline{{\varepsilon_{{{\text{2\_min}}}} }}$ increased with increasing *W*. While no difference was observed in $\overline{{\varepsilon_{{{\text{1\_max}}}} }}$ and $\overline{{\varepsilon_{{{\text{2\_min}}}} }}$ at *W* = 0.49–1.96 N between the two types of sanitary napkin specimens, the absolute values of $\overline{{\varepsilon_{{{\text{1\_max}}}} }}$ and $\overline{{\varepsilon_{{{\text{2\_min}}}} }}$ increased when the low-friction layer was applied at *W* = 1.96–7.84 N. As previously mentioned, Hertz contact theory explains that strain not only increases with *W* but also penetrates deeper into the bulk material as *W* increases^22^. Even if the friction between the two surfaces is assumed to be negligible in Hertz contact theory, the stress at the low-friction layer in the sanitary napkin specimen would increase with increasing *W*. At *W* = 0.49–1.96 N, the stress at the low-friction layer would be smaller than the shear stress of the low-friction layer itself, resulting in no significant difference in $\overline{{\varepsilon_{{{\text{1\_max}}}} }}$ and $\overline{{\varepsilon_{{{\text{2\_min}}}} }}$ and because of the absorption of friction at the low-friction layer. However, at *W* = 1.96–7.84 N, friction at the low-friction layer likely occurred, resulting in a decrease in the absolute values of $\overline{{\varepsilon_{{{\text{1\_max}}}} }}$ and $\overline{{\varepsilon_{{{\text{2\_min}}}} }}$. Notably, the range of *W* where $\overline{{\varepsilon_{{{\text{1\_max}}}} }}$ and $\overline{{\varepsilon_{{{\text{2\_min}}}} }}$ were changed by the low-friction layer aligned with the *W* range where *μ*_d_ decreased because of the low-friction layer.

### Relationship between friction and maximum strain

**Fig. 8:**
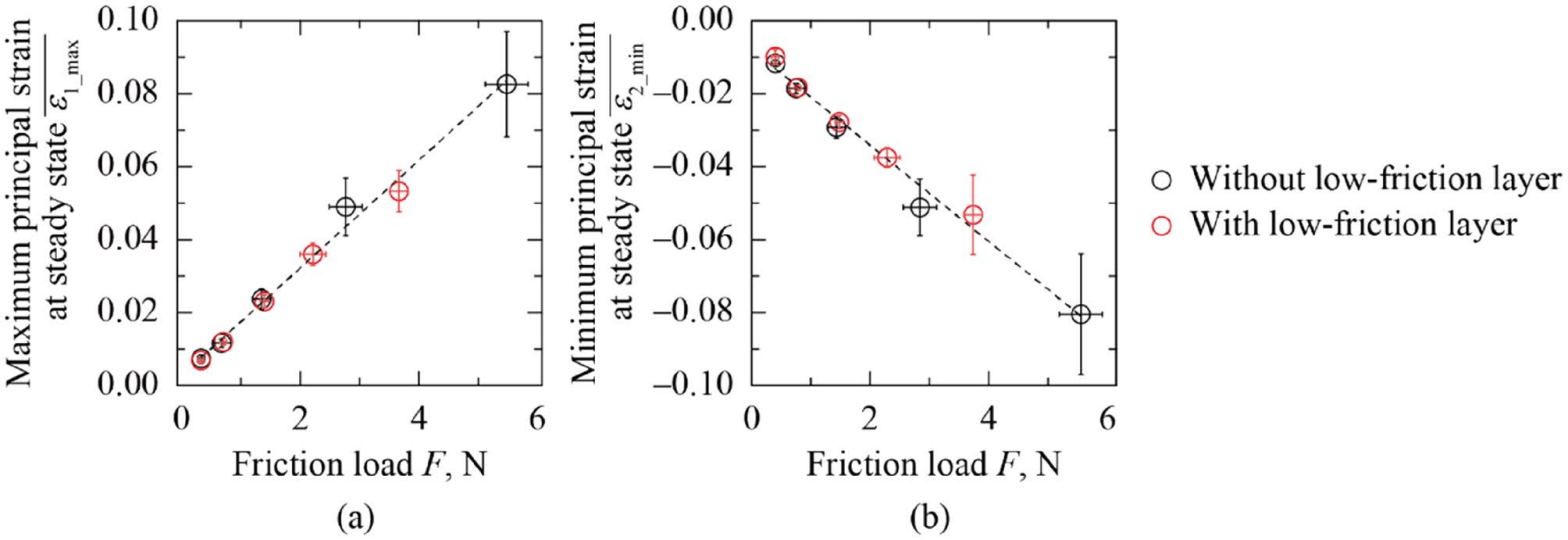
Maximum $\overline{{\varepsilon_{{{\text{1\_max}}}} }}$ and minimum principal strains at steady state $\overline{{\varepsilon_{{{\text{2\_min}}}} }}$ plotted against friction force *F* for the sanitary napkin specimen with and without a low-friction layer

Fig. 8 shows the relationship between the maximum $\overline{{\varepsilon_{{{\text{1\_max}}}} }}$ and minimum principal strain at steady state $\overline{{\varepsilon_{{{\text{2\_min}}}} }}$, and the friction force *F* for the sanitary napkin specimen with and without the low-friction layer. Regardless of the low-friction layer structure, $\overline{{\varepsilon_{{{\text{1\_max}}}} }}$ and $\overline{{\varepsilon_{{{\text{2\_min}}}} }}$ exhibited increasing and decreasing trends toward *F*, respectively. All plots are along the same line, indicating that the lower friction force *F* reduced skin irritation. At *F* < 2 N, corresponding to *W* = 0.49–1.96 N, no difference was observed between the sanitary napkin specimens with and without the low-friction layer. This is because the strain at the low-friction layer is small to generates friction with this layer. However, at *F* > 2 N, corresponding to *W* = 1.96–7.84 N, *F* and the absolute values of $\overline{{\varepsilon_{{{\text{1\_max}}}} }}$ and $\overline{{\varepsilon_{{{\text{2\_min}}}} }}$ decreased when the low-friction layer was applied. This decrease is attributed to the friction at the low-friction layer. These results demonstrate that the low-friction layer in sanitary napkins effectively reduces skin irritation, particularly at high contact pressure. While this finding supports the potential of the low-friction layer to reduce skin irritation under dry conditions, it should be noted that moisture at the skin–material interface can substantially increase friction and alter stress distribution^24,28^. Therefore, future work will extend the present evaluation to moist conditions, including the presence of water or menstrual fluid, to confirm the applicability of these results in more realistic usage environments.

## Limitation

This study has three limitations that should be addressed. First, the influence of menstrual blood on the friction force and strain distribution was not investigated in our experiments. Future research should include friction tests using menstrual blood or equivalent liquids. Second, the friction tests in this study were conducted using an artificial skin block. Although the elastic modulus of the polyurethane gel used in the artificial skin block corresponds to that of human skin in the breast and abdominal areas, it does not replicate the complex stratified structure or the surface frictional behavior of the actual skin in the private zone. This may have influenced the observed strain field distribution, and future studies should include direct comparison with real skin under similar conditions. While literature reports suggest that polyurethane (PU)-based artificial skin models often exhibit friction coefficients comparable to those of human skin, variability among models is considerable, and direct friction and stress distribution measurements for the exact model used in this study are lacking. For example, Nachman & Franklin^28^ demonstrated that a two-layer artificial skin model (PU-based dermis + hydrophilic top layer) mimics both frictional and deformation behavior of human skin under dry and moist conditions. Likewise, Derler et al.^29^ reported that the widely used “Lorica” PU-coated skin equivalent closely matches human skin in terms of friction under dry contact. However, Dąbrowska^30^ cautions that properties such as surface morphology and material composition can lead to significant tribological variance among PU-based models. Furthermore, Shimizu & Nonomura^31^ showed that incorporating microscale surface roughness into PU blocks can enhance tactile and frictional similarity to real human skin. Together, these findings underscore the importance—but also highlight the limitations—of PU as a human skin analog; consequently, our future work will aim to benchmark the exact PU gel model used here against human skin under identical testing conditions. As noted by Morales Hurtado et al.^32^, the sliding friction of artificial skin equivalents can vary markedly under humid conditions, underscoring the need for direct benchmarking with human skin. We plan to obtain such comparative data with human skin in future work to strengthen the tribological relevance of the model. To clarify the effect of the low-friction layer on friction-induced skin irritation, wear tests involving human subjects should be conducted in the future. Third, while the present study focused on mechanical evaluation under controlled laboratory conditions, a large-scale user trial is planned for future implementation. This trial is expected to evaluate the subjective experience of skin discomfort and irritation when using the sanitary product with the low-friction layer, thereby supporting the practical relevance of the current findings.

## Conclusion

The friction test between an artificial skin block and sanitary napkin specimens with and without a low-friction layer was conducted by changing the normal load *W*. The strain distribution on the lateral side of the artificial skin block was measured. The experimental results demonstrate that the dynamic friction coefficient *μ*_d_ decreased with *W* regardless of the napkin specimen structure. However, at *W* = 1.96–7.84 N, *μ*_d_ decreased in the napkin specimen with the low-friction layer. Both the major principal strain *ε*_1_ and the minor principal strain *ε*_2_ reached their maximum tensile and compressive values, respectively, at the center of the bottom half of the artificial skin block. The maximum $\overline{{\varepsilon_{{{\text{1\_max}}}} }}$ and minimum principal strain at the steady state $\overline{{\varepsilon_{{{\text{2\_min}}}} }}$ were measured. The absolute values of $\overline{{\varepsilon_{{{\text{1\_max}}}} }}$ and $\overline{{\varepsilon_{{{\text{2\_min}}}} }}$ increased with *W* regardless of the low-friction layer, but decreased when the low-friction layer was applied at *W* = 1.96–7.84 N. In addition, $\overline{{\varepsilon_{{{\text{1\_max}}}} }}$ and $\overline{{\varepsilon_{{{\text{2\_min}}}} }}$ exhibited positive and negative trends for the friction force *F*. At *F* < 2 N, corresponding to *W* = 0.49–1.96 N, no significant difference in strain was observed between the types of sanitary napkin specimen. However, at *F* > 2 N, corresponding to *W* = 1.96–7.84 N, both *F* and strain decreased in the sanitary napkin specimen with the low-friction layer. This reduction is attributed to the energy dissipation caused by friction at the low-friction layer, which is negligible at lower *W* because of the insufficient stress to overcome the shear stress at the low-friction layer. Considering that the lower the strain on the skin, the lower the skin irritation, particularly under high contact pressure, the low-friction layer in the sanitary napkin effectively reduces skin irritation. However, we acknowledge that this conclusion currently applies only to the dry conditions tested in this study. Future studies will extend these evaluations to moist conditions, including the presence of water or menstrual fluid, and to human wear trials to assess the effect of transport properties and composition changes on friction and stress distribution under more physiologically relevant conditions.

## Methods

### Sanitary napkin specimen

**Fig. 9:**
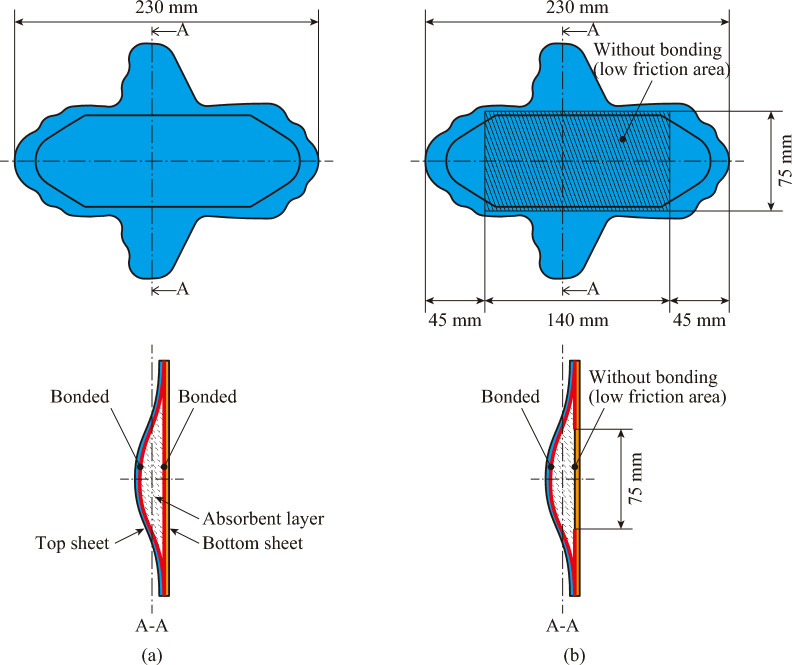
Schematic view of the sanitary napkin specimens.

Fig. 9 shows a schematic of the sanitary napkin specimens. To investigate the effect of a low-friction layer inside a sanitary napkin on skin irritation, two types of sanitary napkin specimens were prepared: a conventional specimen without a low-friction layer and a modified specimen with a low-friction layer. Both specimens were made of the same materials: top sheet, absorbent layer, and bottom sheet. The top and bottom sheets were made of polypropylene nonwoven and nonbreathable polyethylene solid sheets, respectively. The absorbent layer, which was designed to allow menstrual blood to penetrate all materials except the bottom sheet, was made of a superabsorbent fiber nonwoven wrapped in crepe paper. Similar to a commercially available product, the interfaces among the three layers were completely bonded using an adhesive agent for a sanitary napkin specimen without a low-friction layer (Fig. 9(a)). In contrast, the specimen with the low-friction layer (Fig. 9(b)) had a rectangular area (75 × 140 mm) between the absorbent layer and the bottom sheet that was left unbonded. This area corresponded to the area contacting the private areas. This design allows the absorbent layer to move horizontally on the upper surface of the bottom sheet. In a pilot experiment, we confirmed that, under identical contact pressure and sliding velocity conditions, the coefficient of friction on the low‑friction layer (0.279–0.300) was lower than at the interface between the artificial skin block and the top sheet (0.438–0.519). Thus, even though there was no sliding between the top sheet and artificial skin block, friction can occur on the interface between the absorbent layer and bottom sheet, resulting in low friction.

### Artificial skin block

**Fig. 10:**
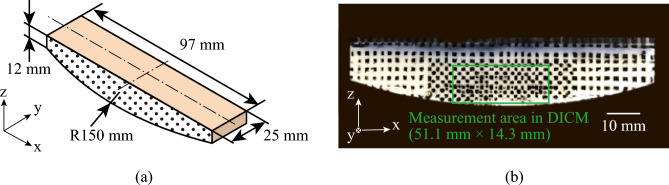
Schematic and image of the artificial skin block.

Fig. 10 shows a schematic and an image of an artificial skin block. The block was prepared using a polyurethane gel (HM01-34002, EXSEAL Co. Ltd., Japan). The elastic modulus of the polyurethane gel was determined by tensile testing to be 62 kPa^33^, which corresponds to that of human skin (specifically, the breast and abdomen)^34^. Polyurethane gel has been used in previous studies as a simplified analog for soft tissue in dynamic contact conditions due to its tunable mechanical and surface properties^28,35^. The polyurethane gel was molded by pouring it into a three-dimensional-printed resin mold along with a curing agent and allowing it to cure for one day at room temperature (Fig. 10(a)). After demolding, the gel surface was covered with a coating agent (HO00-40017, EXSEAL Co., Ltd., Japan), as recommended by the manufacturer, to reduce its inherent tackiness. Without this treatment, the surface was excessively sticky and not suitable for mechanical testing. Since the coating was applied identically to all specimens, it did not affect the comparative evaluation of mechanical or frictional behavior between test groups. To measure the strain distribution on the side surface of the artificial skin block during sliding, black dots were applied using a black marker pen (P-MO-120-MC-BK5, ZEBRA Co., Ltd., Japan).

### Friction test

**Fig. 11:**
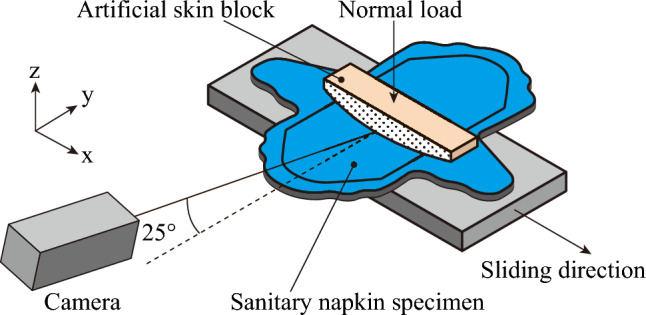
Overview of the friction test.

As shown in Fig. 11, a friction test was performed using a tribometer (*μ*V1000, Trinity Lab., Inc., Japan). The artificial skin block was placed on the sanitary napkin specimen under normal load, with no lubricant applied. After a 0.6-s slide, the friction force was measured at 1000 Hz. The normal loads *W* were set to 0.490, 0.981, 1.96, 3.62, and 7.84 N, respectively. Assuming that the artificial skin block was sufficiently softer than the sanitary napkin specimen and had a Poisson ratio of 0.49, the contact pressure was calculated using Hertz contact theory based on the geometry and elastic modulus of the artificial skin block. The calculated contact pressure ranged from 1.8 to 7.4 kPa. The sliding velocity and distance *d* were set to 5.30 mm/s and 3.18 mm, respectively, corresponding to the friction between the private parts and the sanitary napkin during walking^16^. During the friction test, the side surface of the artificial skin block was observed using a high-speed camera (HAS-L1, DITECT Corporation, Japan) at 8 bit and 100 fps. The camera was set at a 25° angle to the optical path with a resolution of 1280 × 1080 pixels. Considering the angle of the optical path, the practical field of view was 79.4 × 67.3 mm, and the practical pixel size was 62.0 × 62.3 *μ*m. The atmospheric temperature and relative humidity were set to 24.0°C and 37%, respectively.

### Calculation of the strain distribution

To estimate the skin irritation due to friction against the sanitary napkin specimen, the strain distribution on the side surface of the artificial skin block during the friction test was quantified using a digital image correlation (DIC) method with a software (DIPP-Strain, DITECT Corporation, Tokyo, Japan). We focused on the rectangle area (51.1 × 14.3 mm, 821 × 231 pixels) at the center bottom of the side surface (Fig. 10(b)). On the image captured before contact between the artificial skin block and the sanitary napkin specimen, a subset size of 21 × 21 pixels was defined, and each subset was set at every 10 pixels along the *x-* and *z*-axes. The change in the position of each subset was determined based on the intensity distribution^36^. Here, the intensities of *x* = *X* and *y* = *Y* in consecutive frames are denoted as *I*_n_(*X*, *Y*) and *I*_n-1_(*X*, *Y*), respectively. The displacements of the subsets along the *x*- and *y*-axes are represented as *u* and *v*, respectively. The sum of the intensity differences in each subset, *C*(*X* + *u*, *Y* + *v*), was calculated using equation (1).

1 $$C\left( {X + u,Y + v} \right) = \mathop \sum \limits_{i = - M}^{M} \mathop \sum \limits_{j = - M}^{M} \left\{ {I_{n} \left( {X + u + i,Y + v + j} \right) - I_{n - 1} \left( {X + i,Y + j} \right)} \right\}$$

By minimizing *C*(*X* + *u*, *Y* + *v*), *u* and *v* were determined for each subset of each frame. The *u* and *v* values were used to calculate the major and minor principal strains, respectively. Although DIC can theoretically resolve strain on the order of 0.01% under ideal conditions, the accuracy in our experiment may vary depending on the speckle quality, lighting conditions, and deformation complexity of the soft material^37,38^.

## Data Availability

The datasets used and/or analysed during the current study available from the corresponding author on reasonable request.
